# Therapeutic dosing and targeting efficacy of Pt-Mal-LHRH towards triple negative breast cancer

**DOI:** 10.1371/journal.pone.0287151

**Published:** 2023-10-10

**Authors:** Margaret Ndinguri, Lisa Middleton, Jason Unrine, Shu Lui, Joseph Rollins, Emma Nienaber, Cassidy Spease, Aggie Williams, Lindsay Cormier

**Affiliations:** 1 Department of Chemistry, Eastern Kentucky University, Richmond, Kentucky, United States of America; 2 Department of Biological Sciences, Eastern Kentucky University, Richmond, Kentucky, United States of America; 3 Department of Plant and Soil Sciences, University of Kentucky, Lexington, Kentucky, United States of America; 4 Department of Physiology, University of Kentucky, Lexington, Kentucky, United States of America; BRAC University, BANGLADESH

## Abstract

**Objective:**

Pt-Mal-LHRH is a newly synthesized chemotherapeutic agent that was designed to selectively target the luteinizing hormone-releasing hormone (LHRH) receptor expressed by triple negative breast cancer (TNBC). The aim of this study was to evaluate the therapeutic dosing, tumor reduction efficacy, and selective distribution of Pt-Mal-LHRH in-vivo.

**Methods and results:**

LHRH tissue expression levels in-vivo were investigated using western blotting and LHRH was found to be increased in reproductive tissues (mammary, ovary, uterus). Further, Pt-Mal-LHRH was found to have increased TNBC tumor tissue platinum accumulation compared to carboplatin by inductively coupled plasma mass spectrometry analysis. The platinum family, compound carboplatin, was selected for comparison due to its similar chemical structure and molar equivalent doses were evaluated. Moreover, in-vivo distribution data indicated selective targeting of Pt-Mal-LHRH by enhanced reproductive tissue accumulation compared to carboplatin. Further, TNBC tumor growth was found to be significantly attenuated by Pt-Mal-LHRH compared to carboplatin in both the 4T1 and MDA-MB-231 tumor models. There was a significant reduction in tumor volume in the 4T1 tumor across Pt-Mal-LHRH doses (2.5–20 mg/kg/wk) and in the MDA-MB-231 tumor at the dose of 10 mg/kg/wk in models conducted by an independent contract testing laboratory.

**Conclusion:**

Our data indicates Pt-Mal-LHRH is a targeting chemotherapeutic agent towards the LHRH receptor and reduces TNBC tumor growth in-vivo. This study supports drug conjugation design models using the LHRH hormone for chemotherapeutic delivery as Pt-Mal-LHRH was found to be a more selective and efficacious than carboplatin. Further examination of Pt-Mal-LHRH is warranted for its clinical use in TNBCs, along with, other reproductive cancers overexpressing the LHRH receptor.

## Introduction

Identification of new chemotherapeutic compounds that target triple negative breast cancer (TNBC) is critical for enhancing patient outcomes and survival rates. About 10–20% of all breast cancers are considered TNBC, in which, cells lack the expression of the estrogen, progesterone, and human epidermal growth factor receptor 2 (HER2/neu) proteins [1]. Due to a lack of hormonal receptor expression, TNBC is rendered unresponsive to many categories of chemotherapy and hormone therapy medications [1]. Subsequently, TNBC is considered the most aggressive and invasive form of breast cancer, leading to high rates of metastasis beyond the breast and the highest rate of recurrence within the first five years after diagnosis [2]. Moreover, women with TNBC have the highest mortality rates within the first five years of diagnosis with a 77% survival rate for TNBC compared to 93% for other types [3]. TNBC is also regarded as the most prevalent form found in young women aged 20–34 years, contributing to 56% of African American and 42% of white women breast cancer cases [4].

The platinum family of chemotherapeutics have been a predominant treatment option for patients afflicted with reproductive cancers including breast. Platinum compounds are a treatment option for TNBC including the use of carboplatin as a neoadjuvant chemotherapy regimen [5]. Platinum-based drugs elicit cytotoxicity through forming DNA adducts containing covalent interactions that inhibit DNA replication, transcription, and cell division [6] In comparison, cisplatin is known to have a lower mean lethal dose (LD_50_) and smaller therapeutic window than carboplatin; which requires monitoring of a lower dosing regimen due to the increased toxicity [7]. Due to the lack of specific cancer cell targeting, these compounds produce severe side effects including ototoxicity, gastrointestinal toxicity, tubular necrosis, oxidative stress, inflammation, apoptosis, myelosuppression, neurotoxicity, nephrotoxicity, and nausea [6, 8]. Subsequently, neurotoxicity is the dose-limiting factor that results in dose reduction regimens for patients. Another complicating issue is the development of drug resistance mechanisms to cisplatin that have been shown to occur in cancer cells [9, 10]. To attenuate the side effects and possible development of drug resistance, drug discoveries have turned to using novel conjugated approaches to delivering platinum to cancer cells.

One such approach is to overpower resistance mechanisms by increasing intracellular concentration of the drug by targeting cancer cell-specific receptors. Luteinizing hormone-releasing hormone (LHRH), also referred to as gonadotropin releasing hormone, is a decapeptide regulatory hormone involved in reproduction and receptors are found on reproductive tissues [11, 12]. Numerous previous studies have indicated the overexpression of the LHRH receptor in reproductive cancers compared to the corresponding reproductive tissues. Moreover, it has been shown that the LHRH receptor is expressed in normal ovary tissue and is at least 80% lower than in ovarian cancer cells taken from the same patient [13]. One study using radio-imaging with LHRH indicated the radio-receptor marker analysis showed out of 20 cases of breast cancer the positivity rate of the LHRH receptor was 95% and the corresponding normal tissue positivity rate was 20% [14]. Further, s cancer cells with a higher differentiation degree have a higher expression of LHRH receptors, including LHRH receptor overexpression in TNBC [14]. Specifically, about 60% of all human breast cancer cell lines and 74% of TNBC have been shown to express the LHRH receptor [15], making the LHRH receptor a promising therapeutic target [16, 17]. Subsequently, numerous studies have found both the MDA-MB-231 and the 4T1 TNBC cell lines overexpress the LHRH receptor and both cell lines have been amply used to investigate combinational therapy with LHRH. Specifically, both the 4T1 and MDA-MB-231 cell lines have been found to overexpress the LHRH receptor at the protein and mRNA level and are commonly used TNBC cell lines for testing LHRH combination drugs [17, 18]. In addition, database studies including genome and microarray for the LHRH receptor found that 52% of all human breast cancers had binding sites for LHRH [19]. Further, one study examining 17 human TNBC tumor specimens found all samples obtained from the primary tumors were positive for LHRH receptor expression [18]. Moreover, LHRH receptor antagonists have been shown to decrease TNBC cancer cell proliferation in a dose-dependent manner in both in-vitro and in-vivo-xenograft tumor models. Additionally, it is suggested that LHRH receptor is involved in tumor growth and is found more abundantly on advanced stage cancers [18]. Subsequently, the expression of the LHRH receptor was found in some cases to be more enhanced in lymph node metastases compared to primary cancer tumors, indicating the LHRH receptor could be an indication of more aggressive and invasive cancer phenotypes [13].

Various therapeutic modalities have used LHRH conjugation for a targeting approach including curcumin-LHRH, gemcitabine-LHRH, and lytic peptide-LHRH [13, 20]. Further, the conjugates LHRH-prodigiosin and LHRH-paclitaxel have been effective in-vivo at reducing TNBC tumor size [21]. Delivery of platinum encapsulation techniques have been utilized; however, cisplatin loading and unloading has been shown to be difficult [22, 23]. Ndinguri and coworkers have previously reported the design of the compound Pt-Mal-LHRH which was synthezied through performing platinum alterations. The Pt-Mal-LHRH compound was developed by using a malonate linker that conjugated the platinum complex to the peptide LHRH at the D-Lys moiety. Prior findings showed Pt-Mal-LHRH induces cytotoxicity in-vitro in TNBCs by selectively targeting the overexpression of the LHRH receptor and enhances tumor reduction in-vivo [24]. There is a clinical need for advancements in treatment options for individuals afflicted with TNBC, hence, further investigation into Pt-Mal-LHRH for its therapeutic use for TNBC is warranted. In the current study, we examined the therapeutic dosing, efficacy and targeting of Pt-Mal-LHRH to aid in determining its clinical relevance and impact on clinical use and patient care.

## Materials and methods

### Cell culture

The mouse tumor cell line 4T1 was purchased from American Type Culture Collection (ATCC). The cells were cultured in Dulbecco’s Modified Eagle Medium (DMEM) along with 10% Fetal Bovine Serum, 100 U/mL penicillin and 100 μg/mL streptomycin. They were cultured in a 37ºC tissue culture incubator at 95% humidity and 5% CO_2_ and passaged until the desired number of cells was reached.

### Allograft 4T1 tumor therapeutic dose-range study

Female Balb/c mice, 10wk old were purchased from Jackson Laboratory (Bar Harbor, ME) and housed as previously described by Calderon, et al., 2017 [24]. 4T1 cells (1x10^6^) were suspended in 100uL of DMEM not supplemented with FBS and injected into the right mammary fat pad. After orthotopic tumor initiation (~100mm^3^) the mice were allocated into treatment groups based on equal tumor volumes and treated with Pt-Mal-LHRH (2.5, 5, 10, or 20 mg/kg) by intravenous injection as day 1 and tumor growth was monitored for 7 days after. All treatment doses and volume measurements were conducted for each group at the same time of day during each assessment period. The tumor volumes (mm^3^) were calculated using the formula (width)^2^ X length/2, where the length is the smaller of the two measurements. End tumor volume was calculated, and blood samples were collected by left ventricular puncture. Blood was mixed with EDTA and cell blood count data was collected by Hemavet analysis.

### Allograft 4T1 tumor drug comparison study

Using the mouse breast cancer model described above, mice were treated with carboplatin or Pt-Mal-LHRH by intravenous injection and tumor growth was monitored for two weeks. Molar equivalent dose of 2.5, 5, 10, and 20 mg/kg/wk were used for Pt-Mal-LHRH and carboplatin according to their molecular weights. After 2 weeks, end tumor volume was measured (representative tumor images are displayed in the supplemental material) and platinum tissue content in the tumor along with skeletal muscle was digested in aqua regia, diluted, and analyzed by inductively coupled plasma mass spectrometry (Agilent 7900, Santa Clarita, CA). We used certified platinum standards for external standardization and picked all samples and standards with known concentrations of indium as an internal standard. All analysis included duplicates, matrix spikes and digestion blanks.

### Western blot

Tissues were harvested from 12 wk old female Balb/c mice were snap frozen and stored in 10% TCA with acetone. Tissues were washed with acetone and homogenized in 2% SDS. After lysates were prepared and the proteins were separated by SDS-polyacrylamide gel electrophoresis (SDS-PAGE), transferred to nitrocellulose membranes, which were then western blotted with the following antibodies: LHRH receptor (1:3,000; abcam), b-actin (1:10,000; abcam), respectively. The proteins were quantified using the ECL Clarity Max Western Blotting Substrate (Bio-Rad). The western blot images used for analysis are displayed in the supplemental material. Animal protocols were approved by the Committee of Animal Research Care and Use at Eastern Kentucky University.

### Drug distribution studies

Intravenous injection of molar equivalent dose of 20mg/kg of carboplatin and Pt-Mal-LHRH was administered to the female Balb/c mice. After 2.5 hrs tissues were re-harvested for platinum analysis. A secondary study examining 24hr drug distribution was conducted with NCr nu/nu female mice by Charles River Laboratories (Durham, NC). For both studies the platinum tissue content was analyzed as described above.

### Charles river laboratories MDA-MB-231 xenograft drug comparison study

The MDA-MB-231 mouse tumor model was conducted at Charles River Laboratories for third party validation. Orthotopic tumor initiation was performed in female 12wk old NCr nu/nu mice through mammary pad injection of 5x10^6^ cells. Molar equivalent dose of 10 mg/kg/wk were used for Pt-Mal-LHRH and carboplatin according to their molecular weights. Tumor growth was monitored for 2 weeks or with an endpoint tumor volume of 1500 mm³. All study data points are found in the supplemental material. In addition, all animals were euthanized under isoflurane anesthesia for terminal cardiac puncture blood collection and death assured via a cervical dislocation. The guidance set forth in the AVMA Panel on Euthanasia for all animals was followed. For anesthesia, the animals were anesthetized with isoflurane via induction box. They were maintained via nosecone isoflurane. Throughout the anesthetic period, they were monitored for lack of response to stimuli and appropriate cardiopulmonary function. Further, specific humane and study-derived endpoints were described in the IACUC protocols to ensure the welfare of the animal. If any animal presented with clinical issues or if unexpected outcomes were observed, the animal was referred to the Attending Veterinarian for diagnosis and treatment in consult with the study director. Sample sizes/cage of animals used were determined based off previous in-vivo experimental work with Pt-Mal-LHRH including the sample size needed for sufficient statistical relevance (effect size, drug effect) and level of error during each experiment. Animal protocols and numbers were approved by the Committee of Animal Research Care and Use at Eastern Kentucky University (04–2018) and Charles River Laboratories (990202, 980701).

### Statistics

The data is illustrated as mean ± SEM and statistical analyses were performed using GraphPad, Prism 6 (San Diego, CA). Unpaired t-tests, along with, one and two-way ANOVAs were utilized as appropriate.

## Results

### Pt-Mal-LHRH treatment attenuates 4TI TNBC growth

**Fig 1:**
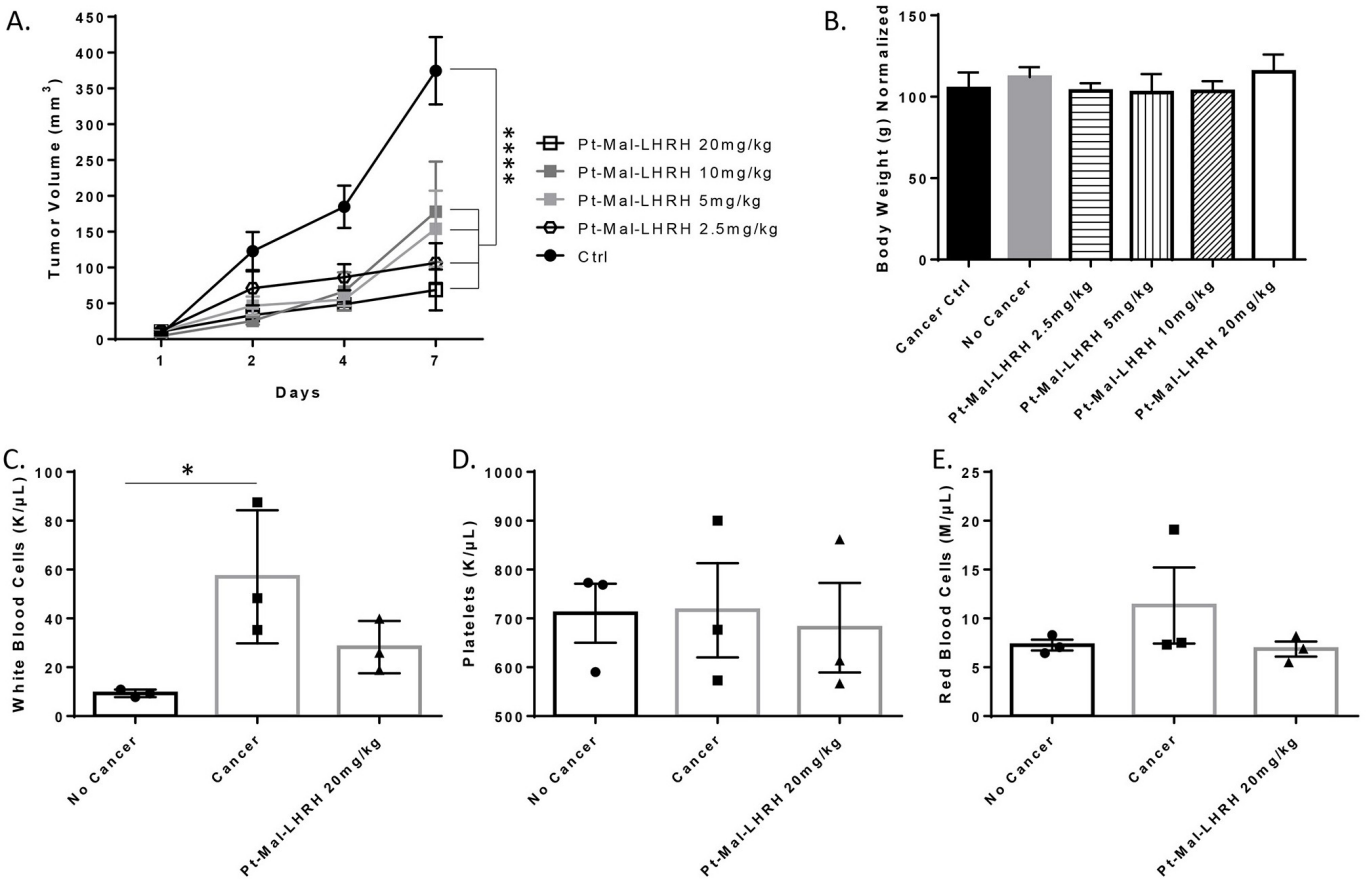
Breast cancer tumor growth is attenuated by Pt-Mal-LHRH treatment. Female Balb/c mice were implanted with 4T1 cells (1x10^6^) in the right second mammary fat pad. Tumors were grown for 7 days, distributed into treatment groups, and treated with Pt-Mal-LHRH intravenously at varying doses. Tumor volume was measured every 3 days and Pt-Mal-LHRH treatments (2.5–20mg/kg) showed significant tumor suppression compared to control growth (A). No decrease in body weight was found with Pt-Mal-LHRH treatments (B). Blood was collected by left ventricular puncture and no difference in white blood cells (C), platelets (D), and red blood cell (E) amounts were found between Pt-Mal-LHRH (20mg/kg) treated and control mice (no cancer and cancer groups). Data is represented as mean ± S.E.M. n = 6 (for each group, A-B), n = 3 (C-E) *p<0.05; ****p<0.0001.

To investigate the safety of therapeutic Pt-Mal-LHRH drug ranges, mice were administered by IV the doses 2.5, 5, 10, or 20 mg/kg. Pt-Mal-LHRH was found to significantly decrease the tumor growth, with 20mg/kg showing the largest amount of tumor regression (Fig 1A). Tumor growth was found to be reduced by 81.7% in response to Pt-Mal-LHRH at the dose of 20 mg/kg (Fig 1A). Further, there was no decrease in mice weight found with Pt-Mal-LHRH dosing indicating drug safety (Fig 1B). In addition, cell blood count analysis showed that Pt-Mal-LHRH administration did not decrease white blood cells, platelets, or red blood cells below normal control, nontumor-bearing mice (Fig 1C–1E). There was a significant increase in white blood cell count in cancer controlled mice compared to the nontumor-bearing mice.

### Pt-Mal-LHRH showed enhanced tumor regression and tumor targeting compared to carboplatin

**Fig 2:**
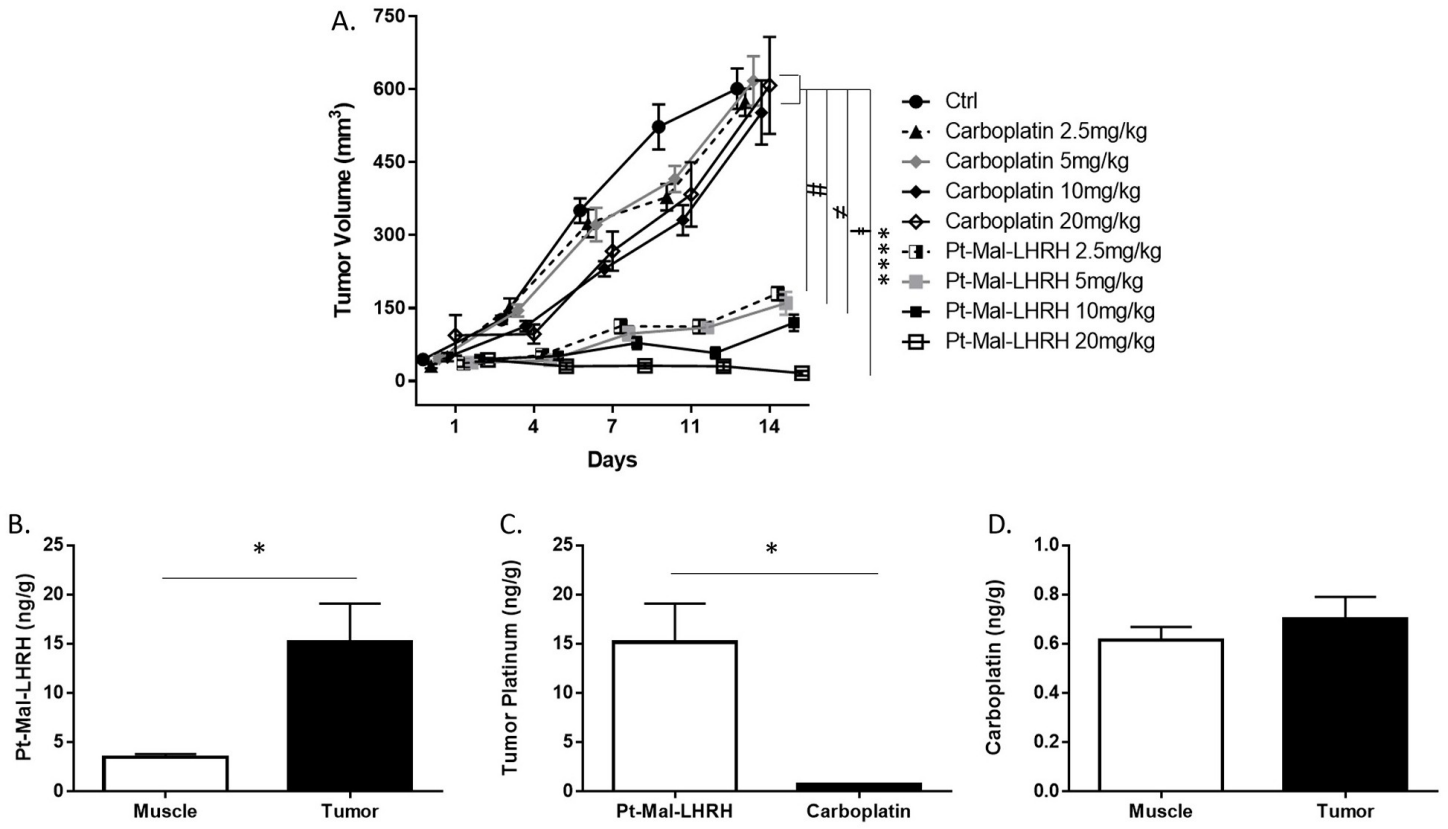
Tumor targeting of Pt-Mal-LHRH attenuated breast cancer tumor growth. Female Balb/c mice were implanted with 4T1 cells (1x10^6^) in the right second mammary fat pad. Tumors were grown for 7 days, distributed into treatment groups, and treated with carbo (carboplatin), or Pt-Mal-LHRH intravenously at varying doses (mg/kg/wk). Tumor volume was measured over 2 wks and Pt-Mal-LHRH doses were found to significantly decrease tumor growth compared to carboplatin and control growth (2A). Pt-Mal-LHRH tumor accumulation was measured by inductively coupled plasma mass spectrometry and found to be significantly higher compared to skeletal muscle (B) and carboplatin (C). Tumor tissue targeting was not found with carboplatin treatment (D). Data is represented as mean ± S.E.M. n = 9 (for each group, A), n = 4 (B-D) * p<0.05, ^#^p<0.0001 (2.5mg/kg Pt-Mal-LHRH compared to ctrl and each carboplatin groups), ^≠^ p<0.0001 (5mg/kg Pt-Mal-LHRH compared to ctrl and each carboplatin groups), ⱡ p<0.0001 (10mg/kg Pt-Mal-LHRH compared to ctrl and each carboplatin groups) **** p<0.0001 (20mg/kg Pt-Mal-LHRH compared to ctrl and each carboplatin group).

To determine the effectiveness of our Pt-Mal-LHRH compound we compared it to carboplatin since the drugs are structurally similar. The previous therapeutic dose ranges were used since no significant toxicological side effects were found. Pt-Mal-LHRH showed a significant attenuation in tumor growth compared to carboplatin and normal control growth (saline), with the largest attenuation found with Pt-Mal-LHRH 20mg/kg treatment (Fig 2A). Each Pt-Mal-LHRH treatment group (2.5–20mg/kg) showed a significant attenuation in tumor growth compared to both the control group and all carboplatin treatment groups. In addition, tumor growth was found to be reduced by 97.5% in response to Pt-Mal-LHRH 20 mg/kg treatment, whereas carboplatin 20 mg/kg only led to a 14.8% reduction (Fig 2A).

**Fig 3:**
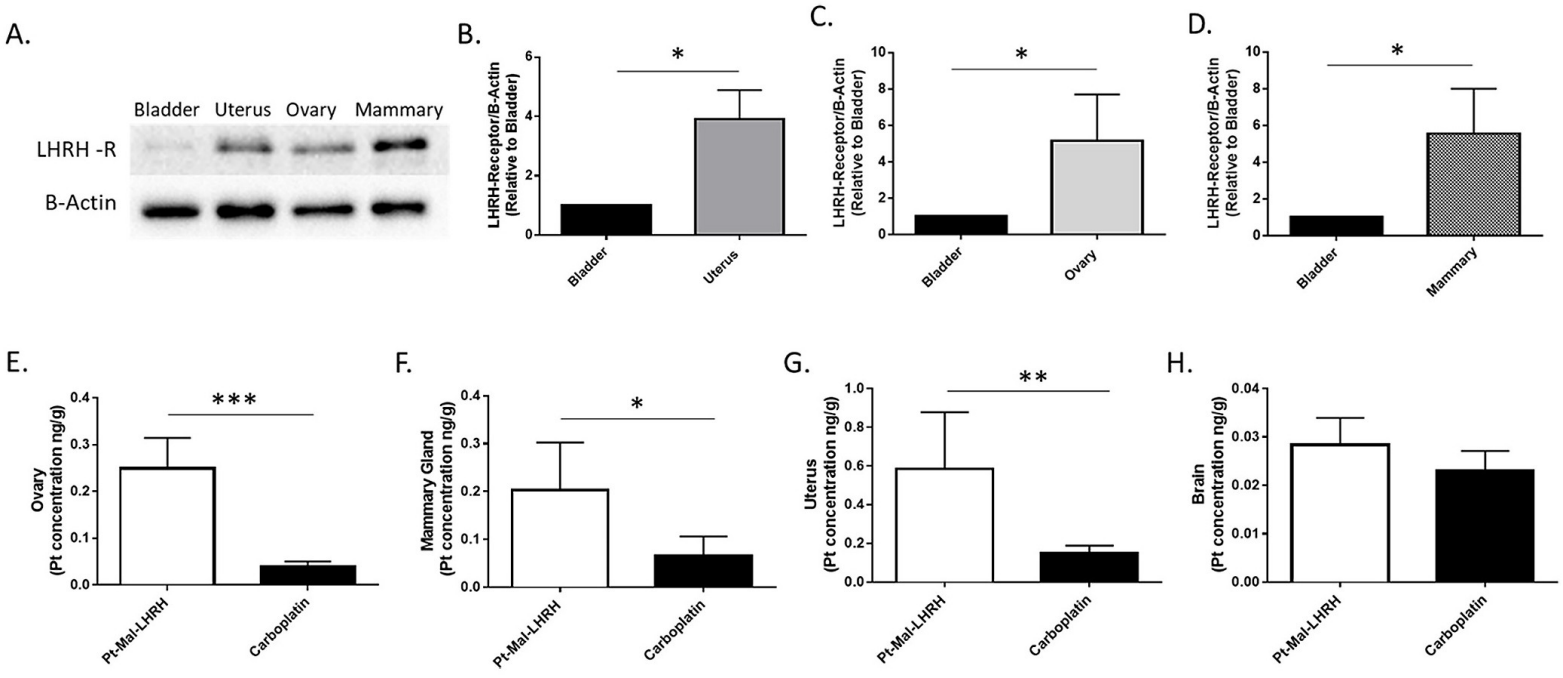
Tissue targeting was found with Pt-Mal-LHRH. Tissues were harvested from untreated 12 wk old female Balb/c mice and LHRH receptor protein was analyzed via western blotting. Increased LHRH receptor expression was found in reproductive tissues compared to normal bladder tissue control, with a significant increase found in mammary, uterine and ovarian tissue (A-D). Female Balb/c mice were injected with 20mg/kg by tail vein injection and tissues harvested after 2.5 hrs. Enhanced reproductive tissue accumulation was found with Pt-Mal-LHRH treatment compared to carboplatin (E-G). No difference in brain accumulation was found (H). Data is represented as mean ± S.E.M. n = 3 (A-D); n = 4 (E-H), *p<0.05, **p<0.01, ***p<0.001.

To examine the tumor targeting ability of LHRH the platinum concentration was examined in the tumor and skeletal muscle (control tissue). Pt-Mal-LHRH was found to have increased accumulation in the tumor tissue compared to skeletal muscle, indicating tumor targeting (Fig 2B). Moreover, Pt-Mal-LHRH showed increased tumor accumulation and targeting ability compared to carboplatin (Fig 2C and 2D). The targeting ability of Pt-Mal-LHRH is due to the LHRH receptor expression, which is known to be increased in reproductive areas. To verify prior work, we also investigated the LHRH receptor expression level in the reproductive tissues specifically in the Balb/c mice. There was increased receptor expression in the reproductive tissues compared to the non-reproductive bladder control, with a significant increase found in the mammary, uterine andovarian tissues (Fig 3A–3D). Acute biodistribution of Pt-Mal-LHRH was investigated and found to have increased tissue accumulation in the mammary, ovaries, and uterus compared to carboplatin (non-receptor targeting; Fig 3E–3G). Brain (Fig 3H) and skeletal muscle (Fig 2D) tissues were used as distribution control tissues and no difference in tissue accumulation was found between Pt-Mal-LHRH and carboplatin treatment. This data indicates that Pt-Mal-LHRH could be considered as a targeting compound towards tumors occurring in reproductive tissues.

### MDA-MB-231 tumors showed decreased growth with Pt-Mal-LHRH treatment

**Fig 4:**
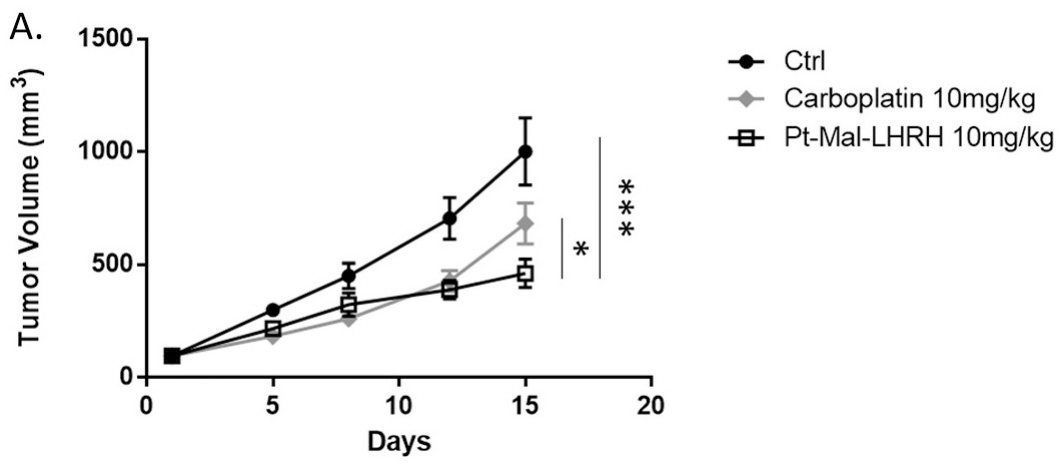
MDA-MB-231 breast cancer tumor growth is attenuated by Pt-Mal-LHRH treatment. Female NCr nu/nu mice were implanted with MDA-MB-231 cells (5x10^6^) in the mammary fat pad by Charles River Laboratories. Mice were placed into treatment groups of Pt-Mal-LHRH, carboplatin or saline (ctrl) and treated intravenously with molar equivalent doses of 10mg/kg/wk. Tumor volume was measured over 2 wks and Pt-Mal-LHRH doses were found to significantly decrease tumor growth compared to carboplatin and control growth. Data is represented as mean ± S.E.M. n = 8 (for each group), *p<0.05, ****p<0.0001.

**Fig 5:**
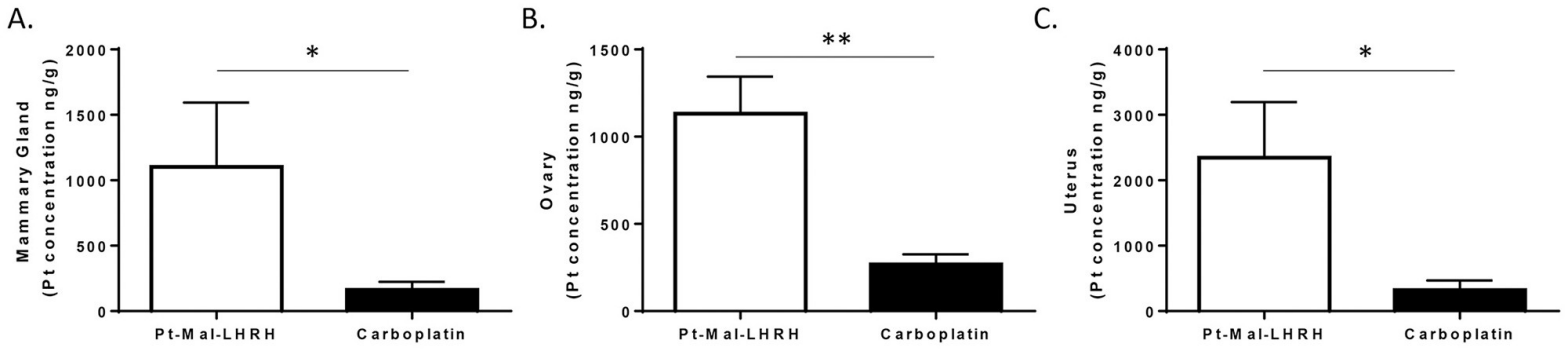
Tissue targeting was found in NCR nu/nu mice. Female NCr nu/nu mice were injected with Pt-Mal-LHRH or carboplatin (10mg/kg) by tail vein injection at Charles River Laboratories. After 24 hrs tissues were harvested and enhanced reproductive tissue platinum accumulation (mammary, ovary, and uterus) was found with Pt-Mal-LHRH treatment compared to carboplatin (A-C). Data is represented as mean ± S.E.M. n = 4 (A-C), *p<0.05, **p<0.01.

To provide in-vivo tumor regression data, validation with a second TNBC cell type (MDA-MB-231) was investigated. Further, an external third party (Charles River Laboratories) was used to conduct the in-vivo tumor study. Since MDA-MB-231 are human derived, the NCr nu/nu immunocompromised mice were used and a dose of 10mg/kg was administered to ensure no toxicity was reached. Pt-Mal-LHRH was found to significantly decrease MDA-MB-231 tumor growth compared to carboplatin (Fig 4), supporting 4T1 tumor study results (Fig 1). The MDA-MB-231 tumor growth was found to be reduced by 54.1% in response to Pt-Mal-LHRH 10 mg/kg treatment, whereas carboplatin 10mg/kg led to only a 32% reduction (Fig 4). Moreover, increased Pt-Mal-LHRH accumulation was found in the mammary, ovaries, and uterus of the NCr nu/nu mice compared to carboplatin, supporting our previous targeting results of Pt-Mal-LHRH in Balb/c mice (Fig 5). In addition, to expand on our existing acute distribution data we examined the biodistribution after 24 hrs (Fig 5). Again, accumulation of Pt-Mal-LHRH was significantly greater in the ovaries, mammary glands and uterus compared to carboplatin.

## Discussion

The design and synthesis of Pt-Mal-LHRH was developed to enhance the potency and accumulation of platinum within tumors overexpressing the LHRH receptor. Our prior work showed Pt-Mal-LHRH enhanced cytotoxicity at a cellular level due to receptor targeting, along with examining tumor regression through administration of an initial single dose [24]. The current study aimed to expand on our understanding of the Pt-Mal-LHRH compound through investigating the therapeutic dose-range and targeting ability. LHRH is a reproductive hormone that plays a critical role in the hypothalamic-pituitary-gonadal axis and aids in regulating reproduction, puberty, and the release of gonadotropins, through binding to the LHRH receptor in tissues [25]. The expression of the LHRH receptor throughout the body has been previously documented and shown to be expressed at higher levels in reproductive tissues including the mammary, uterus, and ovary [11, 12]. Our data also supports increased LHRH receptor expression in the reproductive tissues (Fig 3). In addition, our biodistribution studies showed basal tissue from non-tumor bearing Balb/c and NCr nu/nu mice had enhanced Pt-Mal-LHRH uptake supporting increased LHRH receptor expression in these reproductive tissues (Figs 3 and 5).

Additionally, cancers afflicting reproductive areas including breast, prostate, ovarian, etc,, have been shown to overexpress the LHRH receptor, along with the TNBC cell lines 4T1 (mouse mammary carcinoma) and MDA-MB-231 (human mammary adenocarcinoma) [16, 17, 26–29]. Moreover, the 4T1 and MDA-MB-231 cell lines were chosen for their highly tumorigenic phenotype which enables them to be easily transplantable for primary tumor development in drug studies. Consequently, the thoroughly recognized overexpression of the LHRH receptor in these reproductive cell lines and tumors makes the LHRH hormone an ideal candidate for conjugation in the development of targeting chemotherapeutic compounds.

Our Pt-Mal-LHRH compound design utilizes the LHRH hormone for the selective targeting of the LHRH receptor while reducing its systemic distribution and healthy cell targeting. Further, the cytotoxic effect of Pt-Mal-LHRH was previously shown to be induced by cellular entry of the Pt-Mal-LHRH compound binding to the LHRH receptor and a 20-fold increase in cellular uptake of Pt-Mal-LHRH was found compared to carboplatin due to overexpression of the LHRH receptor in 4T1 cells [24]. Others have also used LHRH as a targeting hormone for chemotherapeutic delivery and found enhanced 4T1 tumor regression, however, they have differed in the modality of delivery and conjugation [17]. The structure of Pt-Mal-LHRH allows the targeting hormone LHRH to be fully accessible for receptor binding for efficient delivery of platinum. Further, the mode of inducing cytotoxicity in cancer cells is directed by the platinum molecule that is incorporated into Pt-Mal-LHRH. Platinum is known to induce DNA damage by forming DNA adducts with preferential binding to guanine sequences in the order of GG>>AG>GA>GXG (X = an undefined residue) [30, 31]. In addition, through utilizing LHRH as the targeting hormone in Pt-Mal-LHRH we were able to show in-vivo distribution to the tumor, in which, there was increased tumor cell uptake/accumulation of the Pt-Mal-LHRH compared to skeletal muscle (Fig 2). Another, platinum compound that is routinely used for chemotherapy treatment is carboplatin. Carboplatin is a nonselective targeting compound, and we found no difference in tissue accumulation of carboplatin in the tumor compared to the skeletal muscle. Further, as a distribution control, the brain tissue was examined and no differences in the minimal accumulation of Pt-Mal-LHRH or carboplatin was found (Fig 3). This indicates the similar chemical structure of each compound limits their entry across the blood-brain barrier. Moreover, while carboplatin is known to have a reduction in toxicity in comparison to cisplatin (another platinum drug family member), its non-selective compound formulation should be improved upon to further its safety in patients. As previously reported, Pt-Mal-LHRH was synthesized containing a platinum compound with a similar chemical structure as carboplatin and has the potential to have even lower side effects than carboplatin due to receptor targeting and selective distribution [24]. In comparison, the leaving groups in carboplatin and Pt-Mal-LHRH are bidentate dicarboxylate ligands, whereas cisplatin has two chloride ligands. The differences in toxicity between the compounds is attributed to the differences in the leaving groups, since a dicarboxylate ligand is a poorer leaving group than the chloride of cisplatin, thus yielding a lower rate of reactivity [32–34].

Further, due to the tumor targeting ability of Pt-Mal-LHRH we found a significant decrease in tumor development in both our dose-range and carboplatin comparison studies. These experiments provided an important deeper examination of the therapeutic and safety profiles of Pt-Mal-LHRH. The investigated doses were chosen based on the therapeutic and toxicology profiles of carboplatin and cisplatin, since the formation of Pt-Mal-LHRH is a combination of two moieties, LHRH and activated cisplatin. Cisplatin is known to have a small therapeutic window with toxicity induced at 2.5–3 mg/kg and embryonic lethality at 5.24 mg/kg in mice, in contrast, carboplatin is known to be less toxic and can be used at higher doses up to 50 mg/kg [35–38]. Both, cisplatin and carboplatin are non-selective targeting chemotherapeutics; thus, Pt-Mal-LHRH has the potential to have enhanced safety in comparison. The end synthesized chemical structure of Pt-Mal-LHRH resembles the less toxic carboplatin while also using LHRH to tailor in-vivo distribution more selectively to the tumor tissues while decreasing excess systemic distribution. There were no indications of toxicological alterations in body weight found with Pt-Mal-LHRH treatments (2.5–20mg/kg), and no decreases in cell blood count data below normal noncancer bearing controls (20mg/kg treatment; Fig 1). Subsequently, the cancer control mice showed a significant increase in the white blood cell count compared to the non-tumor bearing mice, which can be found in certain cancer types [39, 40]. Moreover, carboplatin has a longer half-life than cisplatin and approximately 77% of the drug is excreted through the urine at 24hrs [41]. Subsequently, during our distribution studies we determined that Pt-Mal-LHRH has sustained reproductive tissue targeting over time with increased reproductive tissue accumulation at both 2.5hrs and 24hrs compared to carboplatin (Figs 3 and 5). These data suggest that Pt-Mal-LHRH has both a safety and distribution profile that should be investigated for use in other reproductive cancers afflicting these tissues.

Regarding the tumor reduction efficacy of Pt-Mal-LHRH, there was a significant reduction in tumor development across the three independent mice studies conducted. In the therapeutic dose-range there was a significant decrease in 4T1 tumor volume across the 2.5–20mg/kg doses of Pt-Mal-LHRH (Fig 1). These doses were then compared to carboplatin in a tumor efficacy study and Pt-Mal-LHRH was found to significantly reduce the 4T1 tumor volume compared to carboplatin (Fig 2). Calderon, et. al., previously reported a single dose of 10mg/kg/wk that reduced tumor volume in comparison to carboplatin in 4T1 tumors. Additionally, they showed Pt-Mal-LHRH had increased TNBC cell uptake through binding to the LHRH receptor in-vitro [24]. These data provide a foundation into the possible targeting and distribution in-vivo for our current dosing in our 4T1 tumor studies. Subsequently, to validate our laboratory’s tumor efficacy study, Charles River Laboratories was employed to conduct a secondary study using the MDA-MB-231 human derived TNBC model (Fig 5). In support, Pt-Mal-LHRH was also confirmed using this model to be more efficacious than carboplatin at reducing tumor development.

Together, these data deliver further insights into the efficacy and targeting of Pt-Mal-LHRH as a treatment for TNBC by showing tumor reduction in two separate tumor models. The developmental purpose of Pt-Mal-LHRH was to provide a selective tumor targeting drug paradigm that would target tumors overexpressing the LHRH receptor while mitigating side effects commonly arising from chemotherapy. Our data indicate that drug conjugation using the LHRH hormone could be beneficial for drug delivery and modeling for chemotherapy treatment. Future studies are needed to further examine the toxicology and metabolism of the compound, including, the ability of Pt-Mal-LHRH to target other reproductive cancers overexpressing the LHRH receptor.

## Conclusions

Our results demonstrate that our synthesized Pt-Mal-LHRH chemotherapeutic compound can effectively target and attenuate TNBC tumor growth. This was validated by using two individual TNBC cell line tumor models. Further assessment of Pt-Mal-LHRH treatment showed no decrease in body weight or blood cell count below normal ranges due to toxicity. In addition, the distribution and tissue accumulation results showed targeting of areas overexpressing the LHRH receptor, thus, indicating selectivity towards these tissues while decreasing systemic accumulation, which could potentially mitigate side effects. Taken together, Pt-Mal-LHRH showed enhanced accumulation in reproductive tissues including the ovary, uterus, and mammary compared to carboplatin and effectively targeted TNBC tumors. The tumor targeting of Pt-Mal-LHRH led to selective tumor accumulation upon administration and enhanced attenuation of tumor growth compared to carboplatin. Subsequently, the results of our study support further investigation into the possible clinical use of Pt-Mal-LHRH for patients afflicted with triple negative breast cancer.

### Limitations

While conducting in-vivo experimentation there can be issues that arise including injection error or reaching the designated tumor volume endpoint that can lead to study limitations and decreased statistical power. Injection errors were noted to take place in one animal in the 2.5mg/kg Pt-Mal-LHRH group in Fig 1 and one 20mg/kg animal for the first dose for Fig 2, along with, three 20 mg/kg animals and two 10 mg/kg animals when receiving the second dose. In addition, for Fig 4 two saline animals were removed from the study for reaching the tumor volume endpoint; no injection errors were noted. Further, future in-vivo studies are needed to fully understand the relationship/effect of Pt-Mal-LHRH on the tissue expression level of the LHRH receptor.

## Supporting information

S1 File Contains all data for Figs 1–5 including western blot and tumor images. (DOCX)

S2 File ARRIVE guidelines checklist. (PDF)
