# Supplementary material for: Therapeutic dosing and targeting efficacy of Pt-Mal-LHRH towards triple negative breast cancer
Source: PLoS One. 2023 Oct 10;18(10):e0287151. doi: 10.1371/journal.pone.0287151 (PMC10564129; doi:10.1371/journal.pone.0287151)
Supplement: S1 File — (DOCX) [file pone.0287151.s001.docx]

Supplemental Material

Figure 1A Data Set: Tumor Volume mm^3^

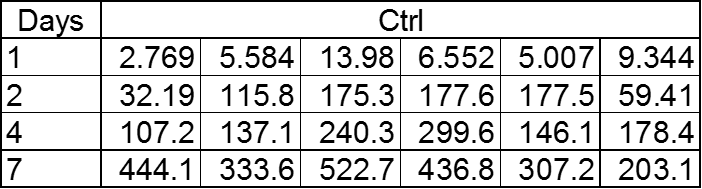


Figure 1B Data Set: Mice Weight (g)

Figure 1C Data Set:

Figure 1D Data Set:

Figure 1E Data Set:

Figure 2A Data Set: Volume mm^3^


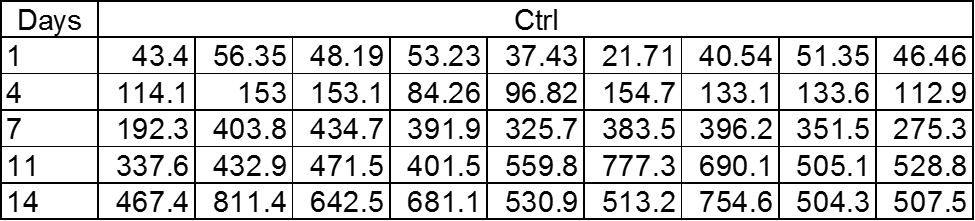

Figure 2A Representative Tumor Imaged Sets:


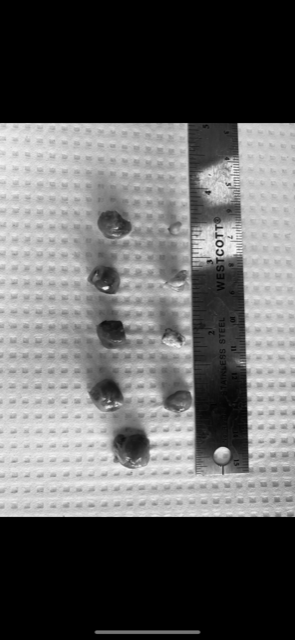


**Control**

**Carboplatin**

**Pt-Mal-LHRH**

**2.5 mg/kg**

**5 mg/kg**

**10 mg/kg**

**20 mg/kg**

Figure 2B Data Set:

|  |  |
| --- | --- |

Figure 2C Data Set:

Figure 2D Data Set:

Figure 3A Western Sets:


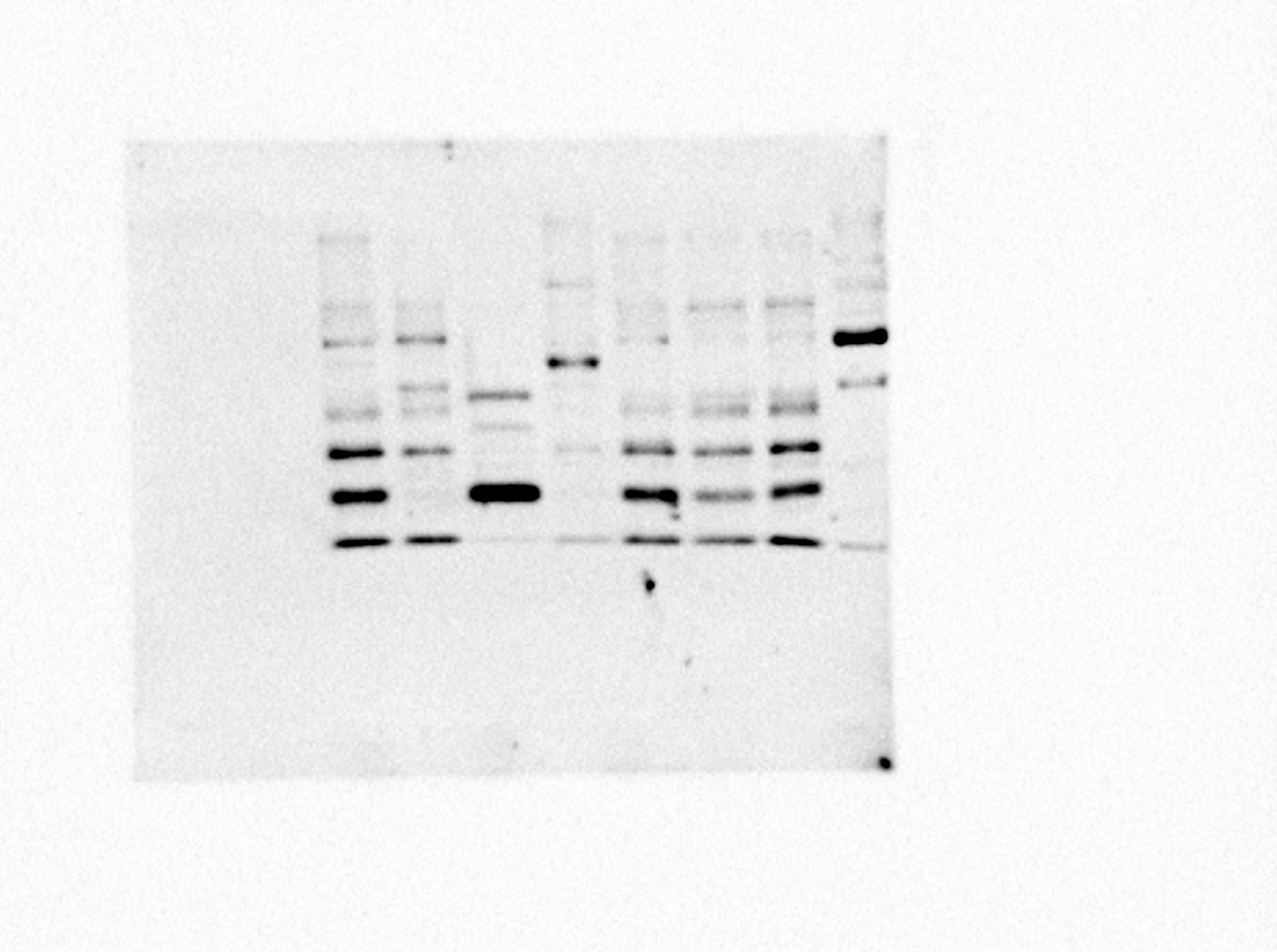

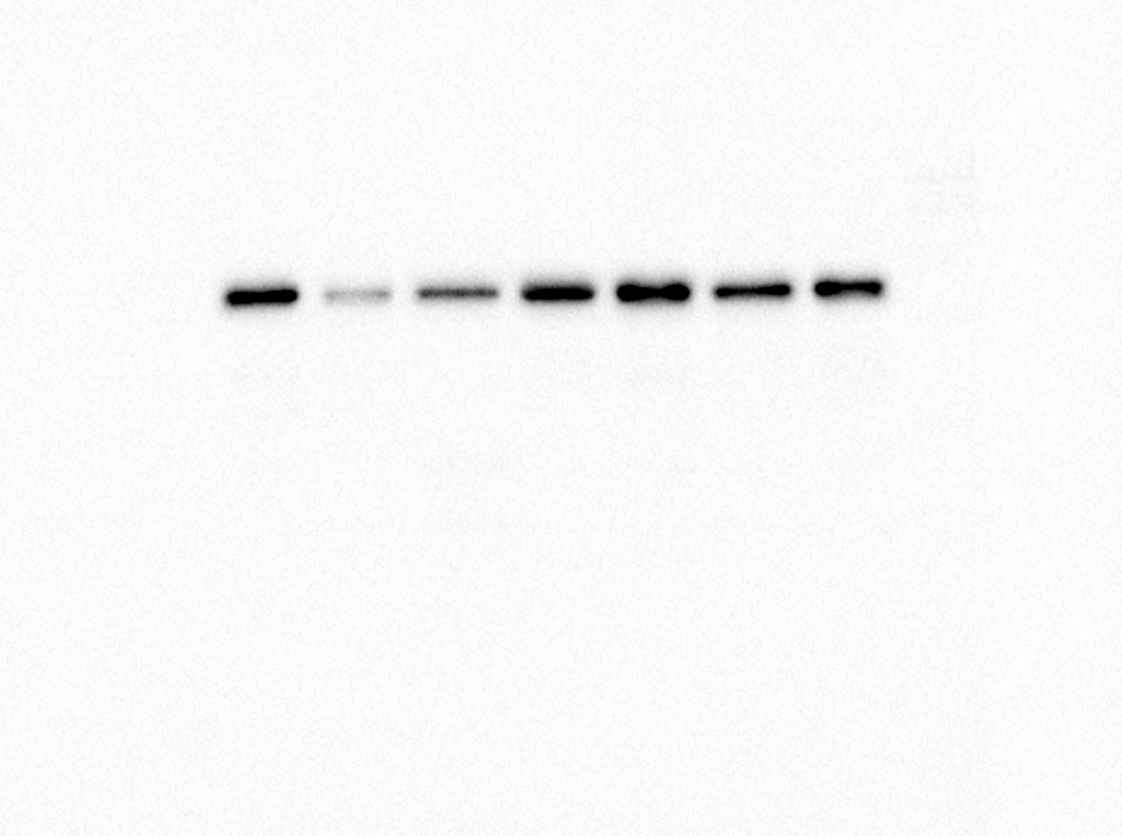


LHRH -R

B-Actin

Bladder Uterus Ovary Mammary


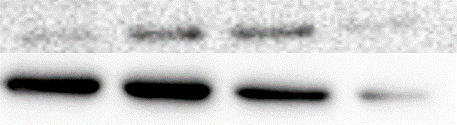


LHRH -R

B-Actin

LHRH -R


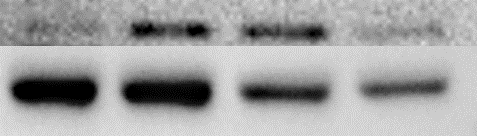


B-Actin

Figure 3B Data Set:

| **Bladder** | **Uterus** |
| --- | --- |
| 1.0 | 5.20 |
| 1.0 | 1.98 |
| 1.0 | 4.53 |

Figure 3C Data Set:

| **Bladder** | **Ovary** |
| --- | --- |
| 1.0 | 7.82 |
| 1.0 | 2.75 |
| 1.0 | 4.89 |

Figure 3D Data Set:

| **Bladder** | **Mammary** |
| --- | --- |
| 1.0 | 5.97 |
| 1.0 | 2.19 |
| 1.0 | 7.75 |

Figure 3E Data Set:

Figure 3F Data Set:

Figure 3G Data Set:

Figure 3H Data Set:

Figure 4 Data Set: Tumor Volume mm^3^

Figure 5A Data Set:

| Pt-Mal-LHRH (ng/g) | Carboplatin (ng/g) |
| --- | --- |
| 298 | 201 |
| 2301 | 295 |
| 299 | 151 |
| 1514 | 0 |

Figure 5B Data Set:

| Pt-Mal-LHRH (ng/g) | Carboplatin (ng/g) |
| --- | --- |
| 1030 | 439 |
| 1736 | 243 |
| 1019 | 195 |
| 743 | 193 |

Figure 5C Data Set:

| Pt-Mal-LHRH (ng/g) | Carboplatin (ng/g) |
| --- | --- |
| 2600 | 535 |
| 2245 | 226 |
| 4342 | 299 |
| 189 | 227 |
